# Oral posaconazole and bronchoscopy as a treatment for pulmonary mucormycosis in pediatric acute lymphoblastic leukemia patient: A case report

**DOI:** 10.1097/MD.0000000000024630

**Published:** 2021-02-12

**Authors:** Yan Liang, Xing Chen, Jinrong Wang, Chunyan Guo, Fengqin Liu, Juan Yang

**Affiliations:** Department of Pediatric Respiratory, Shandong Provincial Hospital Affiliated to Shandong First Medical University, Jinan, Shandong, China.

**Keywords:** acute lymphoblastic leukemia, fiberoptic bronchoscopy, pediatric cancer, posaconazole, pulmonary mucormycosis

## Abstract

**Rationale:**

Mucormycosis is a rare fungal infection that typically occurs in immunosuppressed patients following chemotherapy or hematopoietic stem cell transplantation.

**Patient concerns:**

An 11-year-old child with newly developed acute lymphoblastic leukemia suffered from the paroxysmal left chest pain, fever, and hemoptysis.

**Diagnoses:**

We made a histopathologic diagnosis aided by bronchoscopy techniques, which indicated invasive fungal hyphae that are characteristic of mucormycosis.

**Interventions:**

The patient was treated with oral posaconazole and repeated bronchoscopy interventions for 4 months.

**Outcomes:**

The patient's clinical signs and symptoms and signs were no longer present. The prior lung lesions were also no longer observable using radiologic methods, and a 3-month follow-up with the patient showed no signs of mucormycosis recurrence. Finally, the patient was cured, when the cancer chemotherapy was stopped. Close follow-up for another 2 years showed no evidence of recurrence.

**Lessons:**

Mucormycosis diagnosis is difficult as clinical and imaging findings vary. This case demonstrates that posaconazole monotherapy combined with bronchoscopy interventions may be a safe and effective treatment option for pediatric pulmonary mucormycosis.

## 1 Introduction

Mucormycetes belong to class Zygomycetes, the order Mucorales, subphylum Mucoromycotina. Rhizopus species are the most common organisms isolated in children.^[1]^ Mucormycosis is an opportunistic infection caused by fungi from the order Mucorales, that has an acute onset, rapid progression, high mortality, and overall poor prognosis. Mucormycosis is often classified by the affected organ system, with 6 forms that include rhinocerebral, pulmonary, cutaneous, gastrointestinal, and disseminated mucormycosis, along with uncommon presentations.^[2]^ Pulmonary mucormycosis is the second most common form and recently increases in incidence of mucorales infections have been reported.^[3]^ However, studies of pediatric patients with mucormycosis are limited. In this report, we present a case of pulmonary mucormycosis in an 11-year-old boy diagnosed with acute lymphoblastic leukemia. The purpose of this study is to characterize the presentation, treatment, and follow-up of pulmonary mucormycosis. The patient was successfully treated with oral posaconazole combined with fiberoptic bronchoscopy, which we believe may be effective in other pediatric pulmonary mucomycosis patients as well.

## 2 Case report

**Figure 1:**
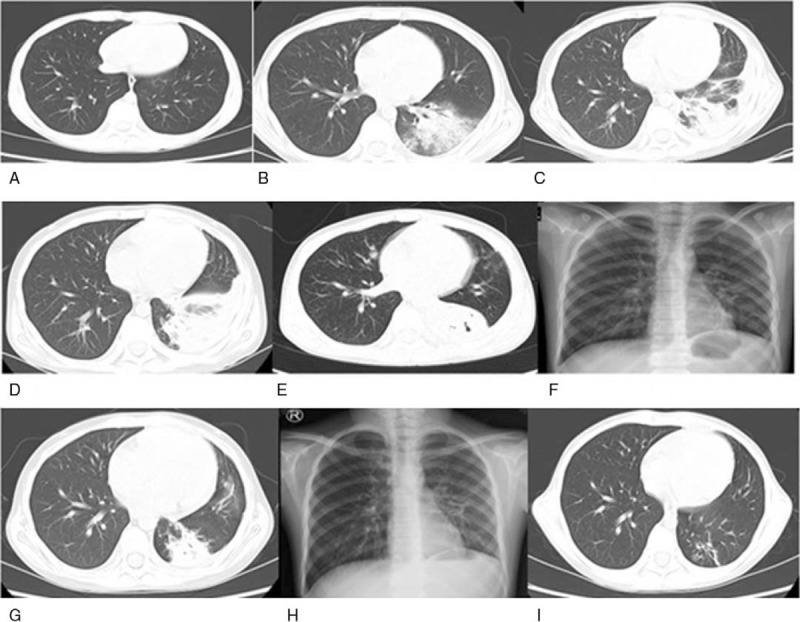
Chest CT findings in the whole process. A, On admission. B, Hemoptysis: pleural effusion with high-density areas in the left lower lobe. C, Voriconazole intravenously for 2 weeks. D, Voriconazole for 4 weeks. E, Aggravation of consolidation, atelectasis, necrosis, and cavitation in the lower left lobe after voriconazole for 12 weeks. F, Oral posaconazole for 1.5 months. G, Oral posaconazole for 3 months. H, Oral posaconazole for 4 months. I, The lesions were obviously absorbed after posaconazole withdrawal for 3 months. CT = computed tomography.

**Figure 2:**
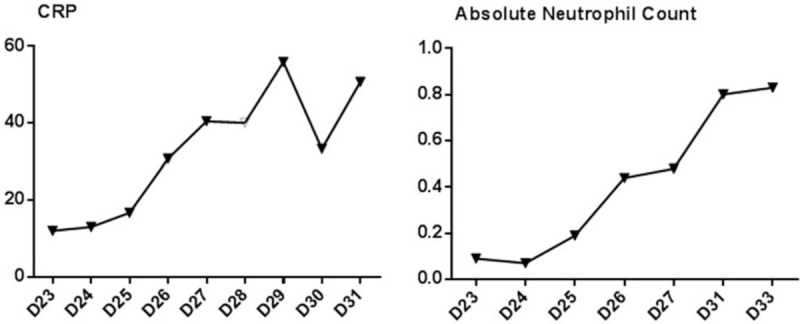
Changes on bacterial infection marker: CRP and absolute neutrophil count. CRP = C-reactive protein.

**Figure 3:**
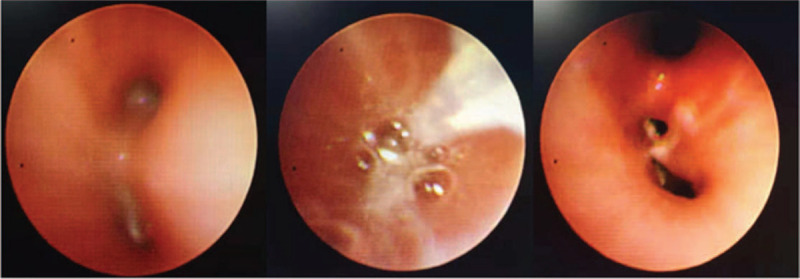
The first bronchoscopy manifestation: severe tracheitis, a lot of sputum and granulation tissues proliferated in the basal segment of the lower left lobe.

An 11-year-old boy was diagnosed with intermediate-risk acute lymphoblastic leukemia (ALL) based on bone marrow morphology, immunology, cytogenetics, and molecular biology examinations. Remission induction therapy was initiated in accordance with the Chinese Children's Leukemia Group (CCLG) ALL 2008 protocol (CCLG-ALL 2008). Chest computed tomography (CT) findings were normal on admission (Fig. 1A). The patient experienced paroxysmal left chest pain on day 23 of combination therapy with vincristine, daunoblastina, L-asparaginase, and dexamethasone, which was exacerbated during hard breathing. On day 25, the patient also developed a fever of 37.8°C with hemoptysis and purulent blood sputum, occurring 1 to 2 times each day, but without dyspnea. Auscultation revealed small, moist rales in the left lobe. Laboratory tests included lower white blood cell count (0.66 × 10^9^/L) with elevated C-reactive protein (CRP; 13 mg/L). Galactomannan, 1,3-β-D-glucan, repeat blood, and sputum cultures were negative. Chest CT images revealed a pleural effusion with high-density areas in the left lower lobe (Fig. 1B). Ceftriaxone was started empirically for 5 days, but did not result in clinical improvement. Absolute neutrophil count and CRP changes were assessed to monitor disease progression. Despite treatment, the patient's fever and CRP level continued to rise in the week following ceftriaxone initiation (Fig. 2). To identify potential sites of hemoptysis and identify the pathogen, bronchoscopy was performed on day 30. We found copious sputum and granulation tissue in the basal segment of the lower left lobe (Fig. 3). Bronchoalveolar lavage (BAL) fluid was harvested for bacterial and fungal cultures. Standard pathogen tests for identifying common bacteria, mycoplasma, tuberculosis, and fungi were uniformly negative. Although a significant increase in fever and CRP suggested the presence of a bacterial infection, we expected that after chemotherapy, depressed immune function would increase the likelihood of opportunistic pathogen infection. We hypothesized that the patient had invasive fungal disease, consistent with the findings of febrile neutropenia and the radiologic findings of pleural effusion with high-density areas. We then initiated diagnostic-driven antifungal treatment on day 31. The patient was taken off ceftriaxone and instead given imipenem/cilastatin, vancomycin, and voriconazole for 2 weeks. The temperature and CRP soon normalized and the chest pain with hemoptysis also subsided, leading us to discontinue imipenem/cilastatin and vancomycin on day 45. The patient continued to receive voriconazole for 10 weeks, intravenously for the first2 weeks and orally for 8 more weeks. The vincristine, daunoblastina, L-asparaginase, and dexamethasone anticancer regimen was continued concurrently during this period of antifungal treatment.

**Figure 4:**
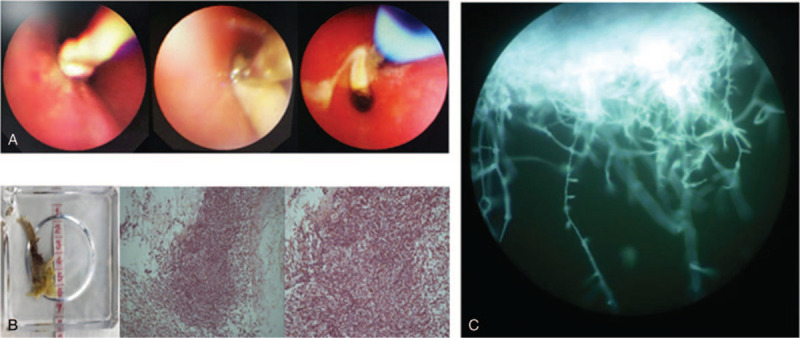
The second bronchoscopy interventions and histopathology. A, The lumen of the left lower lobe was blocked. B, Multiple necrotic tissues were removed and pathological findings indicated mucor mycelia and spores (HE ×200). C, Direct microscopy of mucor mycelia, nonseptate/pauci-septate, ribbon-like hyphae (×400).

**Figure 5:**
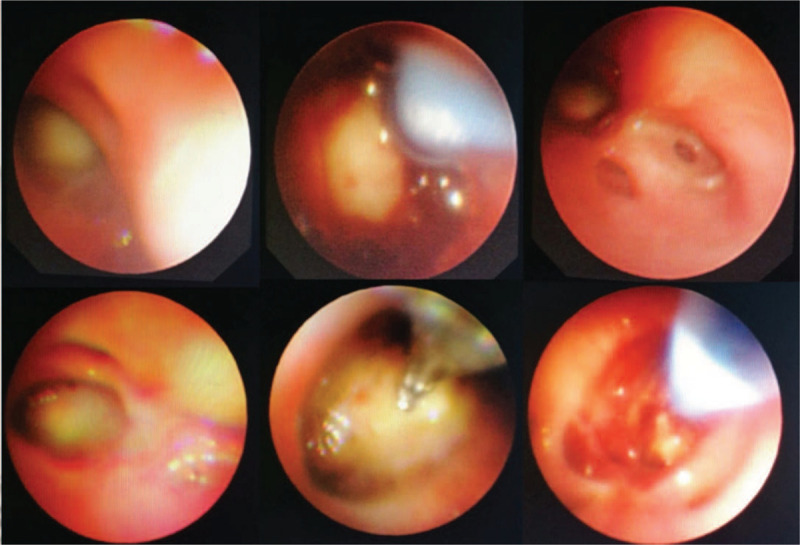
The third and fourth bronchoscopy interventions. Persistent mucous plugs were removed via bronchoscopy techniques, such as biopsy forceps, protected specimen brush, and cryotherapy.

Although respiratory symptoms disappeared in the following 3 months, chest auscultation revealed a weak left lung respiratory murmur. Follow-up CT scans also demonstrated an aggravation of consolidation, atelectasis, low-density lesion necrosis, and cavitation in the lower left lobe (Fig. 1, C–E). These findings suggested empirical therapy was not effective at eliminating the pathogen, despite resolution of the patient's symptoms. A second bronchoscopy was considered for microbiologic and pathologic examinations and for resolving the patient's atelectasis. On day 120 we found on bronchoscopy examination that the left main bronchus was completely blocked and all segments of the left lower lobe collapsed. BAL, biopsy forceps, a protected specimen brush, and cryotherapy techniques were performed to remove multiple necrotic tissues from the bronchial lumen (Fig. 4A), the largest piece being approximately 6 × 2 cm (Fig. 4B). Subsequent histopathology specimens demonstrated the necrotic fibrous tissue had numerous mucor mycelia and spores with characteristic features of (Fig. 4B). Broad, non-septate, irregular (i.e., ribbon-like) hyphae with right-angle branching mycelia were also found in BAL fluid smear under direct microscopy (Fig. 4C). These findings confirmed a diagnosis of pulmonary mucormycosis. Surgical lobectomy and intravenous amphotericin B were recommended; however, the patient refused these treatment options. Oral posaconazole suspension (20 mg/kg daily, given orally in 4 divided doses) was selected as a salvage therapy on day 125. The patient was encouraged to take the medication with high-fat meals. A third and fourth bronchoscopy were performed to remove retained secretions and improve atelectasis. Persistent mucous plugs were removed by bronchoscopy techniques and the patient's atelectasis was almost completely resolved (Fig. 5). Improvement was also noted on imaging after 1.5 months of posaconazole therapy. Regular chest CT or X-ray tests were continued monthly (Fig. 1, F–I). After 4 months on posaconazole, the patient's clinical signs and symptoms and signs were no longer present. The prior lung lesions were also no longer observable using radiologic methods, and a 3-month follow-up with the patient showed no signs of mucormycosis recurrence. The patient achieved complete remission and the chemotherapy was administered as per the protocol. Finally, the patient was cured, when the cancer chemotherapy was stopped. Close follow-up for another 2 years showed no evidence of recurrence.

## 3 Discussion

Although candidiasis and aspergillosis are the most common invasive fungal diseases,^[4]^ high mortality rates and poor prognosis of patients with mucormycosis, in addition to limited proven treatment options, had led to increasing attention to this category of fungal infections.^[5]^ Mucormycosis is found across all age ranges, from newborns to the elderly, and occurs most often in immunocompromised hosts, typically with underlying hematologic malignancies or following hematopoietic stem cell transplant. Other risk factors include neutropenia, broad-spectrum antibiotic therapy, voriconazole prophylaxis, diabetes, and premature birth.^[1,6]^ Accordingly, a retrospective analysis of 4 children with pulmonary mucormycosis showed that 3 patients suffered from diabetic ketoacidosis and 1 had a malignant hematonosis.^[7]^ In the most robust analyses of pediatric mucormycosis involving 226 cases, a 32% mortality rate was reported.^[1]^

Patients with pulmonary mucormycosis typically present with nonspecific clinical findings including fever, cough, chest pain, and hemoptysis. Disease progression often leads to airway obstruction or invasion of adjacent blood vessels, which can cause asphyxia, thrombosis, tissue necrosis, or mass hemoptysis. Radiographic findings are also typically nonspecific for mucormycosis, as pleural effusions, nodules, halo signs, reverse halo signs, and cavities are associated with many types of fungal infections.^[8]^ Our patient only temporarily exhibited fever, chest pain, along with CT findings that included pleural effusions, necrosis, cavities, and atelectasis. Standard laboratory testing did not provide any additional insights, necessitating the use of histopathology, direct microscopy, and culture of the lung lesions. Characteristic mucormycosis hyphae (i.e., broad, hyaline, hyposeptated hyphae) and specific pathologic changes were necessary to make the diagnosis of mucormycosis. Diagnostic methods for mucormycosis infections have advanced in recent years however and now include molecular techniques such as conventional PCR, restriction fragment length polymorphism, DNA sequencing, and real-time PCR.^[9]^ However, the patient refused any of these molecular techniques, as considering the costs.

When treating patients with mucormycosis infections, multiple approaches should be used to first remove exacerbating factors for fungal infection. Surgical debridement and antifungal therapies are considered the cornerstone for mucormycosis treatment^[10–12]^ and prior studies have shown that children treated by antifungal therapy and surgery have a significantly lower case fatality rate.^[13]^ However, according to the ESCMID/ECMM 2019 guidelines, an early complete surgical treatment for mucormycosis, without additional antifungal therapy, is strongly recommended.^[14]^ Both conventional Amphotericin B and AmB lipid complex are also recommended for first-line antifungal monotherapy mucormycosis. However, amphotericin B toxicity can limit its use and manifests as acute infusion-related reactions and dose-related nephrotoxicity.^[15]^ Additionally, when amphotericin B lipid formulations are not available, posaconazole and isavuconazole are strongly suggested as salvage therapy, or in instances of fungal infections that are refractory or intolerant to amphotericin B.^[16,17]^ Prophylaxis should be performed with posaconazole delayed release tablets during remission induction chemotherapy for acute myeloid leukaemia or myelodysplastic syndrome.^[18]^ In these indications, posoconazole has played significant role than amphotericin B. Posaconazole was a new azole antifungal drug. Most available clinical data of posaconazole were in the form of case reports.^[19,20]^ The survival rate of patients who received posaconazole therapy (12/13, 92.3%) was higher than those receiving other antifungals in renal transplant recipients with mucormycosis.^[21]^ Posaconazole was available in oral and intravenous formulations, highly lipophilic, and distributed extensively in tissues. Posaconazole was well tolerated in children with a low incidence of adverse effects than that in adults.^[22]^ General safety and efficacy data were lacking for many antifungal drugs and children are typically treated with off-label antifungal drugs in the absence of established guidelines. Despite currently being unlicensed for use in pediatric patients, 2 oral formulations of posaconazole including a gastro-resistant tablet and an oral suspension have been successfully used to prevent and treat invasive fungal disease.^[22,23]^ To treat systemic fungal infections in children, the recommended posaconazole dose in patients weighing less than 34 kg is 18 to 24 mg/kg daily, given in 4 divided doses. For patients aged 13 years or older or those weighing 34 kg or more, the recommended dose is 800 mg daily, given orally in 4 divided doses. Drug concentrations monitoring is recommended.^[19,24]^ Here in our research, the patient's weight was less than 34 kg; therefore, we selected the posaconazole dose at 20 mg/kg daily. Optimal individualized treatment is also necessary, as disease heterogeneity results in varying disease course and therapeutic response in patients. In our patient, only pulmonary mucormycosis was diagnosed without any other organs affected, such as rhinocerebral, cutaneous, gastrointestinal, and disseminated mucormycosis.

In our patient we also used repeated bronchoscopy techniques which had many diagnostic and therapeutic benefits. BAL and lung tissue removal is known to be beneficial in the diagnosis of many pulmonary diseases.^[25]^ We show here that oral posaconazole plus bronchoscopy intervention techniques can play a vital role in successful pulmonary mucormycosis management, and limits the need for surgical treatment and intravenous amphotericin B. Importantly, this approach was effective for treating the patient and also reduced healthcare-associated costs. Thus, we posit that monotherapy posaconazole with bronchoscopy interventions may be a safe and effective treatment option for pediatric pulmonary mucormycosis patients.

This approach may not be applicable in all situations, as genus and species level identification may sometimes be necessary which requires the use of molecular diagnostic techniques. CT angiography may also be necessary to identify the site and degree of pulmonary thrombosis and embolism. Additionally, once mucor invades major vessels, a surgical lobectomy must be performed to prevent massive hemoptysis and reduce patient mortality.

## Acknowledgments

The authors gratefully acknowledge the Microbiology Laboratory of Shandong Provincial Hospital for their efforts.

## Author contributions

**Data curation:** Yan Liang.

**Investigation:** Yan Liang, Juan Yang.

**Methodology:** Chunyan Guo, Fengqin Liu.

**Resources:** Yan Liang.

**Supervision:** Xing Chen, Jinrong Wang.

**Writing – original draft:** Yan Liang.

**Writing – review & editing:** Xing Chen.

## Abbreviations

Abbreviations: ALL = acute lymphoblastic leukemia, BAL = bronchoalveolar lavage, CRP = C-reactive protein, CT = computed tomography.
